# Multiple functions of the von Willebrand Factor A domain in matrilins: secretion, assembly, and proteolysis

**DOI:** 10.1186/1749-799X-3-21

**Published:** 2008-06-02

**Authors:** Yue Zhang, Zheng-ke Wang, Jun-ming Luo, Katsuaki Kanbe, Qian Chen

**Affiliations:** 1 Division of Musculoskeletal Sciences, Departments of Orthopaedics and Rehabilitation, The Pennsylvania State University College of Medicine, Hershey, Pennsylvania, USA; 2 Cell and Molecular Biology Laboratory, Department of Orthopaedics, The Warren Alpert Medical School of Brown University/Rhode Island Hospital, Providence, Rhode Island, USA; 3 Department of Orthopaedic Surgery, Tokyo Women's Medical University/Daini Hospital, Tokyo, Japan

## Abstract

The von Willebrand Factor A (vWF A) domain is one of the most widely distributed structural modules in cell-matrix adhesive molecules such as intergrins and extracellular matrix proteins. Mutations in the vWF A domain of matrilin-3 cause multiple epiphyseal dysplasia (MED), however the pathological mechanism remains to be determined. Previously we showed that the vWF A domain in matrilin-1 mediates formation of a filamentous matrix network through metal-ion dependent adhesion sites in the domain. Here we show two new functions of the vWF A domain in cartilage-specific matrilins (1 and 3). First, vWF A domain regulates oligomerization of matrilins. Insertion of a vWF A domain into matrilin-3 converts the formation of a mixture of matrilin-3 tetramer, trimer, and dimer into a tetramer only, while deletion of a vWF A domain from matrilin-1 converts the formation of the native matrilin-1 trimer into a mixture of trimer and dimer. Second, the vWF A domain protects matrilin-1 from proteolysis. We identified a latent proteolytic site next to the vWF A2 domain in matrilin-1, which is sensitive to the inhibitors of matrix proteases. Deletion of the abutting vWF A domain results in degradation of matrilin-1, presumably by exposing the adjacent proteolytic site. In addition, we also confirmed the vWF A domain is vital for the secretion of matrilin-3. Secretion of the mutant matrilin-3 harbouring a point mutation within the vWF A domain, as occurred in MED patients, is markedly reduced and delayed, resulting from intracellular retention of the mutant matrilin-3. Taken together, our data suggest that different mutations/deletions of the vWF A domain in matrilins may lead to distinct pathological mechanisms due to the multiple functions of the vWF A domain.

## Introduction

In cartilage, extracellular matrix (ECM) molecules mediate cell-matrix and matrix-matrix interactions, thereby providing tissue integrity. Matrilins (matn) are a novel ECM protein family which consists at least of four members [1]. All the members of matrilin family contain von Willebrand Factor A domains (vWF A domain), EGF-like domains, and a heptad repeat coiled-coil domain at the carboxyl terminus, which is a nucleation site for the oligomerization of the molecule [2,3]. Among the four members, matrilin-1 and matrilin-3 are expressed specifically in cartilage. Matrlin-1 forms a homotrimer and matrilin-3 forms a mixture of homotetramer, -trimer, and -dimer [4,5], in addition to the hetero-oligomers matn-1 and -3 form together [4,6]. It is not known how matn-1 forms a trimer only while matn-3 forms a mixture of tetramer, trimer and dimer. The major structural difference between matn-1 and -3 is that matn-1 contains two vWF A domains while matn-3 contains only one; the second vWF A domain flanking the coiled coil domain is missing from matn-3. In addition, matn-3 contains four EGF repeats, while matn-1 contains only one EGF-like domain. Previously we have shown that the number of the EGF repeats does not affect the assembly of matrilins [4]. In this study, we investigate whether the presence or absence of the vWF A domain adjacent to the coiled-coil is involved in modulating oligomeric formation of matrilins.

The vWF A domain is one of the most widely distributed domains involved in cell adhesion and the formation of multiprotein complexes[7]. These vWF A domain containing molecules include both subunits of the intergrin receptor (α and β), sixteen collagens, and non-collagenous ECM proteins such as matrilins. The property of the vWF A domain in cell adhesion and protein-protein interaction is mediated, in many cases, by the metal-ion dependent adhesion site (MIDAS) located within the domain [8]. We have shown previously that the deletion of the vWF A domain or mutations of the MIDAS motif in MATN-1 abolish its ability to form pericellular filamentous network [9]. This indicates that one of the functions of the vWF A domain of matrilins is to act as an adhesion site for its matrix ligands including collagens and proteoglycans [10,11]. However, this function may not be the only function of the vWF A domain. This is indicated by the recent identification of the mutations of MATN-3 in multiple epiphyseal dysplasia (MED) patients [12].

MED is an osteochondrodysplasia primarily characterized by delayed and irregular ossification of the epiphyses and early-onset osteoarthritis [12]. Two different recessive mutations in the exon encoding the vWF A domain of MATN-3 cause the EDM5 form of MED [12]. These point mutations result in single amino acid changes of V194D or R121W. Subsequent genetic analysis indicates that the R121W mutation is recurrent in multiple families with common or different ancestries [13]. Interestingly, although these residues are conserved in all matrilin family members across species, they are not part of the MIDAS motif [13]. This suggests that these residues in the vWF A domain may play other important roles in addition to protein-protein interactions.

To determine these unknown roles of the vWF A domain in matrilins, we performed a series of deletions and mutations of the vWF A domain in cartilage-specific MATN-1 and -3. We found several novel functions of the vWF A domain of matrilins including regulation of protein secretion, oligomeric assembly, and proteolysis by matrix proteases.

## Materials and methods

### Cloning and Construction of Matrilin-3 cDNAs

Full-length mouse matrilin-3 cDNA was cloned by RT-PCR from the RNA isolated from sternal cartilage of newborn mice. Total RNA was isolated using RNeasy kit (Qiagen). RT-PCR of matrilin-3 mRNA was performed using Titan one tube RT-PCR system (Boehringer Mannheim, Indianapolis IN) according to manufacturer's instruction. In brief, RNA (500 ng), dNTP (0.2 mM/each), DTT (5 mM), RNase inhibitor (5 unit), primers (0.4 μM/each), reaction buffer (1×), and enzyme mix (1 μl) were added in one tube and the volume adjusted to 50 μl. The reverse transcription were performed at 50°C for 30 min and then heated at 94°C for 2 min. Two step-PCR were used in the same tube with the following condition: 94°C 30 sec, 50°C 30 sec, and 68°C 1.5 min for 10 cycles, and then, the annealing temperature was raised to 55°C for another 20 cycles. The nucleotide sequence of matrilin-3 cDNA was confirmed by DNA sequencing. This cDNA and cDNAs encoding chicken matrilin-1 and -3 from previous studies [4], were cloned into an expression vector pcDNA3.1/V5-His (Invitrogen, Carlsbad, CA). Genetic engineering including addition of a N-terminus FLAG tag, addition or deletion of the vWF A domain, and exchange of the coiled-coil domain between MATN1 and MATN3, was performed by overlapping PCR with described primer sets (Table 1). These modified cDNAs were cloned to pcDNA3.1 in a similar fashion. The sequence of all the inserts was confirmed by DNA sequencing.

**Table 1:** Primers used in this study

**Primers**	**Primer Sequences (5'-->3')**	**PCR purpose**	**Results shown in Figures**
1	TAA TAC GAC TCA CTA TAG GG	T7, amplifying inserts from pCDNA3.1	
2	AAG GAC GAT GAT GAC AAA GCT GCA AAT ACA TGT GCA CT	Adding a flag tag	
3	TGT CAT CAT CGT CCT TAT AGT CCC CCC AGA CTC CAC AGC T		
4	GAG GAG AGG GTT AGG GAT AGG CTT A	Amplifying inserts from pCDNA3.1	
5	ACT GCA AGC TGA GCA AGT CTT CTT G	Adding a vWFA domain into minimatn3 (combining with the PCR product of primers 1 and 4)	Figure 4
6	ATCTGC GTT AGA GCC ACA ACA AGC AGT		
7	ACT GCA AGC TGA GCA AGT CTT CTT G	Replacing minimatn3 coiled-coil domain with that of matn1 (combining with the PCR product of primers 1 and 4)	Figure 7
8	ATC TGC GTT AGA GCC ACA ACA AGC AGT		
9	AAA GAA CAA CCT GGG TGG CAG TCA TGA	Introducing R116W mutation in the vWFA domain	Figure 2
10	TCA TGA CTG CCA CCC AGG TGG TTC TTT		
11	GAT GAC AAA GCA CCT CCT CAG CCC AGA	Adding a flag tag	
12	ATC TTC CTC ACT GCA GGT CTT CCC ATC ATT		
13	ACC TGC AGT GAG GAA GAT CCA TGC GAA TGT	Creating Δmatn1 by deleting vWFA2	Figure 5
14	ACC TGC AGT TGC GAA TGT AAA TCT ATA GT		
15	ACA TTC GCA ACT GCA GGT CTT CCC ATC AT	Creating Δmatn1_del by deleting 4 amino acids from Δmatn1	Figure 6
16	ACT TGC TCA GCT TGC AGT GGT GGG TCA		
17	TGT GGC TCT AAC GCA GAT TTT CAT TTG	Amplifying mantn1 coiled-coil domain	
18	ACT TGC TCA GCT GTC AGT GGT GGG TCA		
19	TCT GGC TCT AAC GCA GAT TTT CAT TTG	Amplifying mantn1 vWFA2 domain	

**Figure 1:**
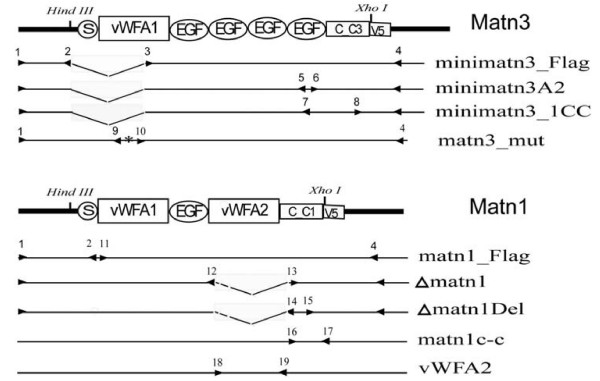
Construct production and primer set. The relative locations of the primers used to produce various MATN1 and MATN3 constructs are shown underneath the schematic models of matrilins. The primers are numbered as in Table 1. S: signal peptide; C-C: coiled-coil domain.

The primers are numbered as in Figure 1.

### Transfection of Matrilin cDNAs

cDNA constructs of matrilin-3 and -1 were transfected into COS-7 cells (Monkey Kidney Fibroblast Cells) or MCT cells (Immortalized Mouse Chondrocytes) [14] using LIPOFECTAMINE (Life technology, Rockville, MD) according to manufacturer instruction. Briefly, COS-7 cells or MCT chondrocytes were trypsinized and counted. Each 60 mm plate were seeded with 6 × 105 cells, with were allowed to attach overnight and reach 70% confluence in DMEM supplied with 10% FBS (Life technology). The following day, the cells were rinsed with DMEM and subjected to a DNA/LIPOFECTAMINE(Life technology) mix for 5–24 hours. Five μg cDNA were used for single transfection and 4 μg/each cDNA were used for co-transfection, respectively. The DNA/LIPOFECTAMINE mixture was aspirated and replaced with 3 ml DMEM supplied with 1% FBS. The media from transfected cell culture were collected at different time points (1, 2, 3, and 4 days) after transfection. Cells were lysed on ice for 10 minutes in a lysis buffer as previously described [15]. Cell lysates were centrifuged at 4°C for 10 minutes. Supernatant of the cell lysate as well as the conditioned medium were analyzed using western blot. Some transfected cells were treated with matrix protease inhibitors including EDTA and actinonin at indicated concentrations for 48 hours before the conditioned medium was collected for analysis.

### SDS-Polyacrylamide Gel Electrophoresis and Western Blot

Western blot analysis was performed with collected conditioned medium or cell lysates from transfected cell culture. For non-reducing condition, collected samples were mixed with standard 2× SDS gel-loading buffer[16]. For reducing conditions, the loading buffer contains 5% b-mercaptoethanol and 0.05 M DTT. Samples were boiled for 10 minutes before loaded onto 10% SDS-PAGE gels, or 4–20% gradient gels as indicated. After electrophoresis, proteins were transferred onto Immobilon-PVDF membrane (Millipore Corp., Bedford, MA) in 25 mM Tris, 192 mM glycine, and 15% methanol. The membranes were blocked in 2% bovine serum albumin fraction V (Sigma Co., St. Louis, MO) in PBS for 30 minutes and then probed with antibodies. The primary antibodies used were a monoclonal antibody against the V5 tag (diluted 1:5000) (Invitrogen), and a monoclonal antibody against FLAG (diluted 1:1000) (Affinity BioReagents). Horseradish peroxidase conjugated goat anti-mouse or goat anti-rabbit IgG (H+L) (Bio-Rad Laboratories, Melville, NY), diluted 1:3,000, was used as a secondary antibody. Visualization of immunoreactive proteins was achieved using the ECL Western blotting detection reagents (Amersham Corp., Heights, IL) and exposing the membrane to Kodak X-Omat AR film. Molecular weights of the immunoreactive proteins were determined against two different sets of protein marker ladders.

### Protein Pulse-Chase

COS-1 or MCT cells were cultured in DMEM + 10% FBS in 12-well plates overnight. Matrilin-3 or MED-mutant matrilin-3 cDNA was transfected into the cells using Lipofectamin 2000 (Invitrogen). Three days after transfection, cells were starved for 2 hours in 0.5 ml cysteine and methionine free medium (Sigma), pulse-labeled in 100 μCi/ml medium of S-35 methionine (Amersham) for 1 hour, and chased in normal medium. After harvest of culture supernatants, monolayer cells were lysed in 1% NP-40, 50 mM Tris, pH 7.4. Immunoprecipitation was carried out by incubating culture supernatant or cell lysate with 1.5 μl anti-V5 antibody (Invitrogen) at 4°C for 2 hours, followed by coupling to protein A/G plus agarose (Santa Cruz) overnight at 4°C. After precipitation, the samples were eluted by boiling after washing 3 times with 0.5% Triton 100 in TBS. The eluted proteins were separated by electrophoresis in a 4–15% SDS-PAGE gel, followed by transferring to a PVDF membrane and exposed to X-ray films.

### Immunohistochemistry

After cells from Cos-1 and MCT cell lines were seeded onto 8-well chamber slides, 1 μg wild-type matrilin-3 or MED-mutant matrilin-3 cDNA was transfected into each well using Lipofectamin 2000 (Invitrogen). Three days after transfection, monolayer cells were fixed with 70% ethanol, 50 mM glycine for 1 hour. Immunofluorescence staining was performed by incubation of anti-V5 primary antibody (Invitrogen) at 1:200 for 2 hours, followed by incubation with donkey anti-mouse rhodamine secondary antibody (Jackson Laboratory) at 1:200 dilutions in the presence of Hoechst Stain Solution (Sigma). Slides were mounted with coverslips in Gel/Mount (Biomed).

## Results

### MED mutation in the vWFA domain of MATN3

**Figure 2:**
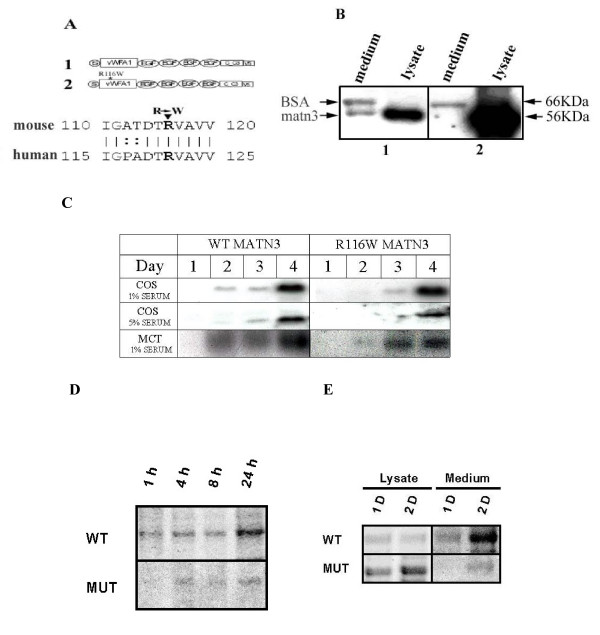
**Secretion of matrilin-3**. A. Schematic diagram of MATN3 constructs. 1: wildtype MATN3, and 2: R116W mutant MATN3. The diagram below indicates the position of the point mutation in mouse MATN3 and its homology to human MATN3; a line indicates identical amino acid residues between mouse and human MATN3 while double dots indicate conserved changes of amino acid residues. B. Western blot analysis of recombinant matn-3. Cell lysate or conditioned medium of COS cells transfected with construct 1 or 2 was collected 48 hours after transfection, separated on a 10% SDS-PAGE under reducing conditions, blotted to a membrane, and incubated with antiserum against the V5 tag. Bound antibodies were detected with a peroxidase-coupled secondary antibody and a chemiluminescence detection kit. Cross-reaction to BSA in the medium samples containing serum is indicated. C. Time course of matrilin-3 secretion. Cos cells or MCT chondrocytes were incubated in the presence of 1% or 5% serum as indicated. Conditioned medium was collected at the indicated days after transfection, and analyzed on a 10% SDS-PAGE under reducing conditions. Western blot analysis was performed with antiserum against the V5 tag of the recombinant matrilin-3. In both COS cells and MCT chondrocytes incubated under different concentrations of serum, the quantity or the speed of the secretion of R116W MATN3 was diminished in comparison to the wildtype MATN3. D. Autoradiograph of matrilin-3 secretion in culture medium of MCT chondrocytes. MCT cells were transfected with wildtype (WT) or R116W mutant (MUT) matrilin-3 cDNA. Synthesized proteins were pulse-labelled with S-35 methionine for 1 hour and chased for 1 hour (1 h), 4 hours (4 h), 8 hours (8 h), and 24 hours (24 h). After each chase period, conditioned medium was collected for immunoprecipitation with an antibody against the V5 tag of the recombinant matrilin-3. Equal protein amount was loaded in each lane of the SDS-PAGE gel for autoradiogram analysis. E. Autoradiograph of recombinant matrilin-3 in the cell lysate and conditioned medium of matrilin-3 cDNA transfected Cos cells. Cos cells were transfected with wildtype (WT) or R116W mutant (MUT) matrilin-3 cDNA. Synthesized proteins were pulse-labelled with S-35 methionine for 1 hour and chased for 1 day (1 D), or 2 days (2 D). After each chase period, conditioned medium was collected and cells were lysed for immunoprecipitation with an antibody against the V5 tag of the recombinant matrilin-3. Equal protein amount was loaded in each lane of the SDS-PAGE gel for autoradiogram analysis.

To understand the structure-function relationship of cartilage-specific matrilins: MATN1 and 3, a series of cDNAs containing mutations and deletions in MATN1 and 3 were constructed (Fig. 1). We first tested whether the MED point mutation (R116W) in mouse MATN3, which is equivalent to the R121W mutation in MED patients, affected synthesis and secretion of matn3 (Fig. 2A). The cDNA harbouring the MED mutation (R116W MATN3) was transfected into Cos cells. Both culture medium and cell lysates from transfected cells were subject to western blot analysis (Fig. 2B). While culture medium from wildtype matn3 transfected cells contained both matn3 (56 KD band) and BSA (66 KD band), the medium from R116W MATN3 transfected cells did not contain matn3. Furthermore, excessive amount of R116W matn3 protein was seen in the cell lysate. This suggests that the matn3 mutant protein was retained inside of the cells, which resulted in defective secretion. To verify this hypothesis, we determined the time course of the secretion of both wildtype and mutant matn3 in culture medium (Fig. 2C). At two days post-transfection, wildtype matn3 was detected in the culture medium but matn3-mut was not. Three days post-transfection, the mutant matn3 started to be detected in the medium. Diminishing quantity and speed of the secretion of mutant matn3 was seen in both transfected Cos cells and MCT chondrocytes (Fig. 2C).

**Figure 3:**
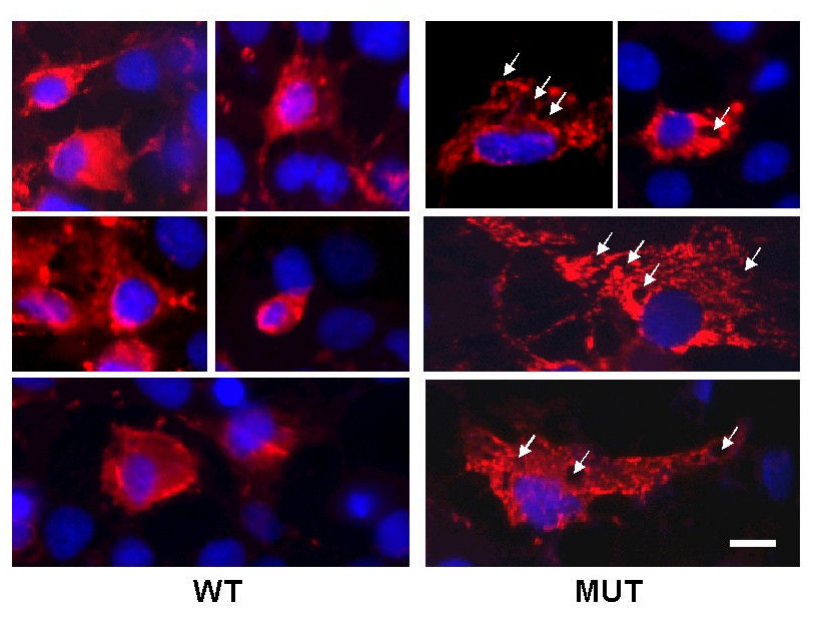
Immunocytochemistry analysis of matrilin-3 transfected cells. Cos cells were transfected with either wildtype (WT) or R116W mutant (MUT) matrilin-3 cDNA. Three days post-transfection, immunocytochemistry analysis was performed with an antibody against the V5 tag of the recombinant matrilin-3. Matrilin-3 positive signals are indicated by rhodamine (red) fluorescence, while the cell nucleus is indicated by Hoechst dye (blue). Please note the expanded cytoplasm in mutant matrilin-3 transfected cells. Arrows indicate the presence of multiple vacuoles in those cells. Bar = 6 μm.

Because the amount of matn3 detected by western blot reflected the accumulation of matn3 due to both matn3 synthesis and degradation, we then chased secretion of radiolabelled matn3 after pulse-labelling its synthesis. Secretion of the mutant matn3 was greatly reduced than that of the wildtype matrilin-3 in MCT chondrocytes (Fig. 2D). In Cos cells, secretion of mutant matn3 was also significantly reduced, and the majority of synthesized mutant matn3 was retained intracellularly (Fig. 2E). Immunocytochemistry of matrilin-3 indicated that numerous vesicles that contained mutant mtn3 were present in the cytoplasm (Fig. 3). In contrast, only few vesicles were present in wildtype matn3 expressing cells. The cytoplasm of the mutant matn3 expressing cells was greatly expanded with multiple vacuoles. Thus, a point mutation (R116W) in the vWF A domain caused a deficiency of matrilin-3 secretion, intracellular retention of the mutant protein, and altered cytoplasm in mutant matrilin-3 expressing cells.

### Insertion of vWFA2 domain into MATN3

**Figure 4:**
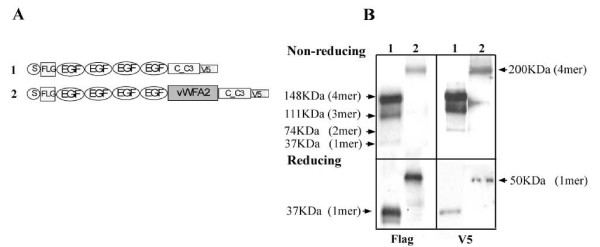
**Insertion of vWF A2 domain into MATN3 alters its oligomerization**. A. Schematic diagram of Construct 1: MINI-MATN-3; and Construct 2: MINI-MATN-3 A2. B. Western blot analysis of the conditioned medium from Cos cells expressing (1) MINI-MATN-3, or (2) MINI-MATN-3, collected 72 hours after transfection. FLAG: analysis using the antiserum against the FLAG tag at the N-terminus of the recombinant matn-3. V5: analysis using the antiserum against the V5 tag at the C-terminus of the recombinant protein. Reducing conditions and the molecular weights of the Mini-Matn3 oligomers were indicated on the left, while the molecular weights of the Mini-Matn3 A2 oligomers are indicated on the right.

To understand whether the vWFA domain plays a role in modulating matn3 oligomeric assembly, we inserted the vWFA2 domain from MATN1 into MATN3, which normally does not contain the vWFA2 domain (Fig. 4A). The secreted matrilin peptides were collected from the medium of transfected cells, and analyzed on a western blot. Anti-Flag was used to detect the Flag tag at the N-terminus of the peptide, and anti-V5 was used to detect the V5 tag at the C-terminus of the peptide. To simplify analysis, we used a mini-matn3, which has the same oligomeric properties as the full-length matn-3 [4]. Like what we showed previously with the full-length matn3, the mini-matn3 formed a tetramer (148 KD), a trimer (111 KD), and a dimer (74 KD) (Fig. 4B, lane 1). In contrast, the vWFA2-inserted mini-matn3 (mini-matn3A2) formed a 200 KD tetramer, but no trimer or dimer (Fig. 4B lane 2). Thus the absence of vWFA2 domain from MATN3 affects its oligomerization.

### Deletion of vWFA2 domain from MATN1

**Figure 5:**
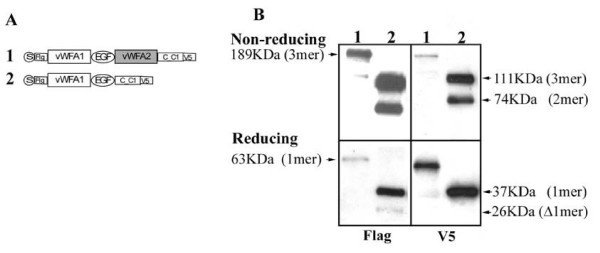
**Deletion of vWF A2 domain from MATN1 alters its oligomerization**. A. Schematic diagram of Construct 1: MATN1; and Construct 2: MATN1ΔA2. B. Western blot analysis of the conditioned medium from Cos cells transfected with Construct 1 or Construct 2, under the same experimental conditions as described in the Figure 3 legend. Reducing conditions and the molecular weights of the Matn1 oligomers were indicated on the left, while the molecular weights of the Matn1ΔA2 oligomers are indicated on the right.

To perform the converse experiment, we deleted the vWFA2 domain from wildtype MATN1 (Fig. 5A). While matn-1 formed a predominant trimer (200 KD) under non-reducing conditions, Δmatn-1 formed a trimer (111 KD) and a dimer (74 KD) (Fig. 5B). Thus, the vWFA2 domain is also important for oligomerization of matn1 oligomers. This conclusion is consistent with our previous observation [4]. Under reducing condition, matn-1 presented a 63 KD monomer only. For Δmatn-1, besides a 37 KD monomer, there was another peptide of 26 KD (Fig. 5B, Flag). This product could not be detected with the V5 antibody directed at C-terminus of coiled-coil (Fig. 5B, V5). This suggests that this peptide is a Δmatn-1 without the coiled-coil domain due to proteolytic cleavage.

### Proteolysis of matn1

**Figure 6:**
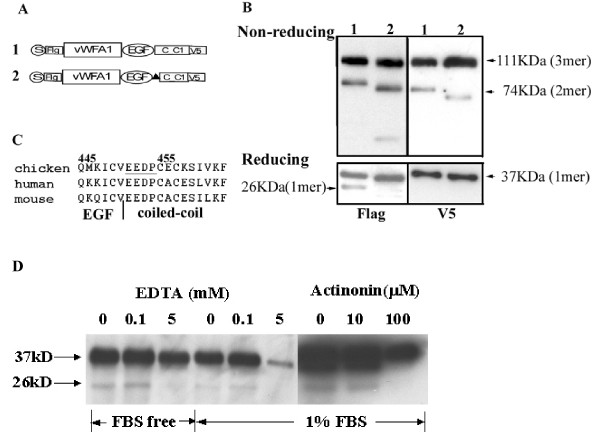
**Deletion of the latent matrix protease site eliminates processing, but does not affect oligomerization of MATN1ΔA2**. A. Schematic diagram of Construct 1: MATN1ΔA2; and Construct 2: MATN1ΔA2Del. B. Western blot analysis of conditioned medium of Cos cells transfected with Construct 1 or 2 under the same experimental conditions as described above. C. Proteolytic cleavage of MATN1ΔA2Del is inhibited by matrix protease inhibitors EDTA and actinonin. Cos cells transfected with MATN1ΔA2Del were incubated with EDTA and actinonin at indicated concentrations for 48 hours in the presence or absence of 1% FBS. Conditioned medium was collected for western blot analysis under reducing conditions using antiserum against the FLAG tag.

The presence of the 26 KD N-terminal peptide fragment suggests that there is a cleavage site at the junction between the vWFA2 domain and the coiled coil domain, which is responsible for matn1 processing. This junction consists of only four amino acid residues EEDP, which precedes the cysteine residues responsible for covalently link matrilin molecules in the coiled-coil domain (Fig. 6C, underlined residues). To test this hypothesis, these four amino acid residues were deleted from the junction site, and the resulting cDNA Δmatn-1Del was transfected into COS cells (Fig 6A). Δmatn-1Del still formed trimer and dimer under non-reducing conditions (Fig. 6B). Thus elimination of the junction site did not affect trimer or dimer formation. However, deletion of the junction site eliminated the 26 KD peptide under reducing conditions, although the 37 KD monomer still existed (Fig. 6B). This suggests the junction site is a proteolytic processing site of matn-1. To further test this hypothesis, we determined whether the presence of the inhibitors of matrix proteases affected the proteolytic processing of matn-1. The presence of 5 mM EDTA in the medium completely inhibited proteolytic processing of matn-1 either in the presence or absence of serum, as did 100 μM actinonin (Fig. 6D). This suggests that cleavage by matrix proteases is responsible for the generation of the 26 KD fragment.

### Exchange of the coiled-coil domain between MATN1 and MATN3

**Figure 7:**
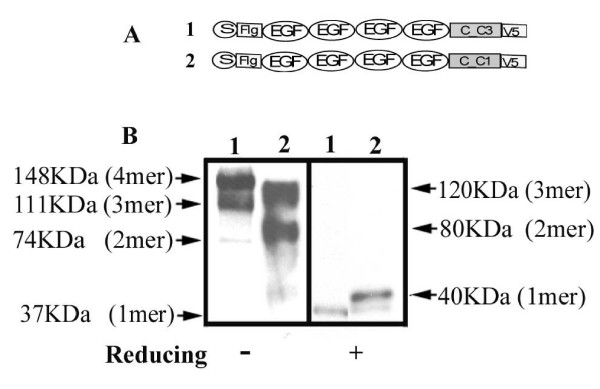
**The coiled-coil domain regulates oligomerization of matrilins**. A. Schematic diagram of Construct 1: MINI-MATN3; and Construct 2: MINI-MATN3_1CC. B. Western blot analysis of conditioned medium collected from Cos cells transfected with Construct 1, or 2 using antiserum against the V5 tag. Reducing conditions are indicated. Molecular weights of the MINI-MATN3 oligomers are indicated on the left, while those of MINI-MATN3_1CC are indicated on the right.

To determine whether the coiled-coil domain also played a role in regulating matrilin assembly, we replaced the coiled coil domain in mini-matn-3 with the coiled-coil domain from matn-1 (Fig. 7A). Instead of a combination of a tetramer, a trimer, and a dimer resulting from homo-oligomerization of the native mini-matn-3 (Fig. 7B, lane 1), the chimeric mini-matn-3 with the coiled-coil from matn-1 formed a trimer and a dimer only, but no tetramer (Fig. 7B, lane 2). Thus, the coiled-coil domain is involved in regulating matrilin oligomeric assembly.

## Discussion

Our study suggests that the matrilin vWF A domain, a widely distributed structural module in integrins and ECM proteins, plays a role in regulating protein secretion, assembly, and proteolysis, in addition to its well-documented role in cell-matrix adhesion [9]. These newly discovered functions of the vWF A domain of matrilins are discussed as follows.

### Secretion

We show that a single point mutation in the vWF A domain of mouse MATN3 (R116W), equivalent to the MED mutation (R121W) in human MATN3, leads to a deficiency of matrilin secretion *in vitro *which is consistent with previous reports[17]. In addition to the decrease of the amount of the mutant protein secreted into the medium (Fig. 2B), the secretion time course is markedly delayed for 24 hours (Fig. 2C, D). In the meantime, excessive amount of the mutant protein is accumulated intracellularly (Fig. 2B, E). These observations indicate that intracellular retention of the mutant protein is responsible for the deficiency of protein secretion in quantity and speed. Consistent with this hypothesis, we observed a great increase of intracellular vesicles that contain mutant matrilin-3 (Fig. 3). The vWF A domain is composed of about 200 amino acid residues arranged into multiple α-β units, which results in a three dimensional structure of a central β sheet core flanked by α helices [8]. Because R121 is located in one of the β strands, despite the molecular mechanism is still under investigation, it strongly suggests that abnormal protein folding contributes to the secretion deficiency of the mutant protein.

Although matrilin-3 is the only matrilin family member that has been associated with chondrodysplasia so far, more and more point mutations within the vWF A domain of matrilin-3 have been reported to cause MED. They include mutations A219D, I192N, T120M, and E134K [13]. Interestingly, all of these MED-causing mutations are located in the β strands in the center of the vWF A domain, which are important for the folding of the protein structure [13]. It suggests that the secretion deficiency due to intracellular retention of the mutant protein, as demonstrated by this study, is a common mechanism of matrilin-3 associated MED. Such mechanism is similar to that of a point mutation of cartilage oligomeric matrix protein (COMP), which also leads to MED or related pseudoachondroplasia[18]. It has been demonstrated previously that the mutant COMP is retained in the rough endoplasmic reticulum [19]. This retention in turn results in excessive accumulation of the proteins that are associated with COMP such as collagen type IX, whose mutation also leads to similar clinical manifestation[20]. Our observation that cells expressing mutant matrilin-3 exhibit expanded cytoplasm with multiple vacuoles, which is similar to the phenotype of mutant COMP expressing cells [18,20], suggests that mutated matrilin-3 or COMP may lead to common cellular phenotype. In light of the recent discovery that COMP interacts with matrilin-1, -3, and -4[21], our finding here lends support to the hypothesis that mutations in any of these interacting proteins including matrilin, COMP, or collagen IX, result in a secretion defect, which manifests in common chondrodysplasia pathological phenotypes. It should also be noted that a portion of the mutant protein is secreted into the medium. However, we do not know whether the mutant protein is defective in its adhesion to matrix ligands or subject to extracellular proteolysis. These possibilities remain to be determined in future studies.

### Assembly

The oligomeric assembly of matrilins is complex. This complexity is two fold. First, in contrast to some ECM protein families such as collagens that always form a trimeric structure, different matrilin member forms different set of oligomers. While the major oligomeric forms of matrilin-1, -2 and -4 are trimers, matrilin-3 is a tetramer [4,22]. Second, in addition to the major oligomeric form, each matrilin has minor oligomeric forms. For example, matrilin-2 has a tetramer and a dimer in addition to a trimer, and matrilin-3 has a trimer and a dimer in addition to a tetramer. So far, two theories have been proposed to explain the cause of heterogeneity of matrilin oligomers. One is proteolytic processing, which proposes that the heterogeneity of the matrilin derives from the proteolytic cleavage of a single matrilin oligomer [22]. Indeed, studies using the peptide of the coiled-coil domain demonstrate that each matrilin peptide forms a single homo-oligomer, with matrilin-1, -2, and -4 being a trimer and matrilin-3 being a tetramer [23,24]. Furthermore, Klatt et al. demonstrated that proteolytic cleavage of a matrilin-4 trimer generates a dimer and a monomer [22]. However, the proteolytic processing theory cannot explain all the heterogeneity of matrilin oligomers. For example, it cannot explain how a matrilin-2 trimer gives rise to a tetramer through proteolytic cleavage.

We proposed an alternative theory that heterogeneity of oligomeric forms of matrilins may arise from imperfect oligomerization [4], in addition to protein processing. The imperfect oligomerization hypothesis was based on the fact that the amino acid sequence of the oligomeric nucleation site coiled-coil domain, although strongly favours one oligomeric form, has ambiguity for alternate forms [25]. This ambiguity is modulated by the vWF A domain next to the coiled-coil domain. Our study here put this hypothesis to test. First, replacing the coiled-coil domain of matrilin-3 with that of matrilin-1 changes the matrilin-3 oligomeric forms from a combination of a tetramer, a trimer, and a dimer into a combination of a trimer and a dimer, reminiscent of those of matrilin-1 (Fig. 7). Thus, the coiled-coil domain primarily determines the oligomeric forms of matrilins. Second, the vWF A domain next to the coiled-coil further modulates the diversity of matrilin oligomeric forms. Deletion of the vWFA2 domain from matrilin-1 converts the formation of a predominant trimer into a mixture of trimer and dimer (Fig. 5), while insertion of the vWFA2 domain into matrilin-3 converts the formation of a mixture of tetramer, trimer, and dimer into a tetramer only (Fig. 4). The vWFA domain may achieve this modulatory role in two ways, by affecting either matrilin processing or assembly. The identification of a latent matrilin-1 cleavage site (EEDP) at the junction of the vWFA2 domain and the coiled-coil domain seems to suggest that different oilgomeric forms of matrilins arise from processing at this site. However, deletion of this cleavage site, which clearly eliminates protein processing, does not reduce the number of different matrilin oilgomeric forms (Fig. 6B). Thus, the diversity of the matrilin oligomeric forms cannot be attributed to protein processing alone. The vWFA2 domain, therefore, must play a role in regulating matrilin oligomeric formation as well.

### Proteolysis

**Figure 8:**
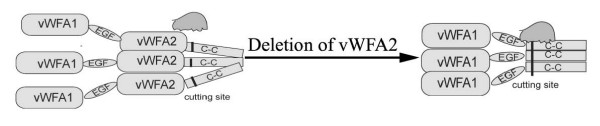
**The junction region of matrilins contains potential proteolytic cleavage sites**. Schematic diagram illustrating that the vWF A2 domain shields the neighbouring cleavage site by steric hindrance.

In this study, we discovered a previously unsuspected latent proteolytic site in matrilin-1 between the vWF A domain and the coiled-coil domain. We found that, although wildtype matrilin-1 is not cleaved, deletion of the vWF A2 domain generates a cleaved peptide without the coiled-coil domain. The cleavage site was predicted to be in the junction region according to the molecular weight of the cleaved peptide. We confirmed the location of the cleavage site by deleting the junction region sequence EEDP, which eliminates processing (Fig. 6B). Based on these findings, we hypothesize that the proteolytic site is normally shielded by the abutting vWF A2 domain because of the extremely short junction region (Fig. 8). Thus the vWF A2 domain may inhibit proteolytic processing of matrilin-1 by steric hindrance of the neighbouring cleavage site. This cleavage site is sensitive to the inhibitors of matrix proteases. Proteolytic cleavage is inhibited by cation chelator EDTA at 5 mM (Fig. 6C). This suggests that this matrix protease is cation dependent. The proteolysis is completely inhibited by 100 μM actinonin, which is known to inhibit 100% of the activity of aggrecanses, but only 23% of the activity of matrix metalloproteinase (MMP) [26]. This indicates that the matrix protease that cleaves mutated matrilin-1 is likely to be a member of aggrecanse family. Further studies are needed to determine the identity of this matrix protease.

Based on our current study and the studies from other laboratories [22,27], we propose that the junction region between the coiled-coil domain and its N-terminal neighbouring domain contains hot spots for proteolytic cleavage by matrix proteases. This region varies in length, ranging from a mere 4 amino acids in matrilin-1 to a unique domain of 72 to 83 amino acids in matrilin-2 (Table 2). For a small junction region such as that in matrilin-1 (4 amino acids), the presence of the neighbouring vWF A2 domain shields the site from being cleaved. On the other hand, the cleavage site in a longer junction region such as that in mouse matrilin-4 (14 amino acids) is not completely shielded by the neighbouring vWF A2 domain [22]. The latent matrilin-1 cleavage site contains two glutamic acid residues. Such a pair of glutamic residues has been identified as a cleavage site in the junction region of matrilin-4 [22], and is present in the junction regions of matrilin-2 and -3. Thus, they are candidate sites of matrilin proteolysis by matrix proteases.

**Table 2:** The junction region of matrilins contains potential proteolytic cleavage sites.

**Matrilin**	**Species**	**Amino Acid Sequence**	**Number (a.a.)**
MATN1	Human	EEDP	4
	Mouse	EEDP	4
	Chicken	EEDP	4
MATN2	Human	KLKKGICEALEDSDGRQDSPAGELPKTVQQPT ESEPVTINIQDLLSCSNFAVQHRYLFEEDNLL RSTQKLSHSTKPSGSPLEE	83
	Mouse	KLKEGICEALEDSGGRQDSAAWDLPQQAHQP TEPEPVTIKIKDLLSCSNFAVQHRFLFEEDN LSRSTQKLFHSTKSSGNPLEE	83
	Chicken	ELKVQICEALRNSAHQQHLSSGRLHRTNPQPSGPESTTVEITDVLACPSLAIQHKYLFEDSQSHSTRTTAKT	72
MATN3	Human	ATEEARRLVSTEDA	14
	Mouse	DIEEARSLISIEDA	14
	Chicken	RATTSSLVTDEEA	13
MATN4	Human	PEEGISAGTELRSP	14
	Mouse	PEEGIGAGTELRSP	14
	Chicken	PEEGRGETEIRSP	13

A pair of glutamic residues as the putative matrix cleavage site is underlined and is present in all the members of matrilin family across species with the only exception of chicken MATN2. a.a.: amino acid.

One of the major functions of the junction region containing these cleavage sites is to process matrilins and generate proteolytic fragments. The cleavage in the junction region of matrilins separates the vWF A domain that binds matrix ligands from the coiled-coil domain that oligomerizes matrilins. Such proteolytic cleavage may destabilize or destroy matrilin filamentous network in extracellular matrix. Our study raises a possibility that mutation/deletion of the vWF A domain may change its conformation to expose the mutant matrilin for accelerated proteolytic degradation.

## Conclusion

Different mutations/deletions of the vWF A domain in matrilins may lead to distinct pathological mechanisms due to the multiple functions of the vWF A domain. This may explain how different mutations within matrilin-3 lead to a variety of cartilage diseases.

## Abbreviations

PAGE: polyacrylamide gel electrophoresis; RT-PCR: reverse-transcription polymerase chain reaction; MED: multiple epiphyseal dysplasia; COMP: cartilage oligomeric matrix protein

## Competing interests

The authors declare that they have no competing interests.

## Authors' contributions

YZ carried out the molecular genetic studies, participated in the sequence alignment, ZW carried out the proteolysis assays, JL participated in the immunocytochemistry, KK participated in the cloning of cDNA constructs, QC conceived the study, and participated in its design and coordination and drafted the manuscript. All authors read and approved the final manuscript.
